# Dietary and Physical Activity Behaviours in African Migrant Women Living in High Income Countries: A Systematic Review and Framework Synthesis

**DOI:** 10.3390/nu10081017

**Published:** 2018-08-03

**Authors:** Lem Ngongalah, Judith Rankin, Tim Rapley, Adefisayo Odeniyi, Zainab Akhter, Nicola Heslehurst

**Affiliations:** 1 Institute of Heath & Society, Newcastle University, Newcastle-Upon-Tyne, Tyne and Wear NE2 4AX, UK; l.n.ngongalah2@newcastle.ac.uk (L.N.); judith.rankin@newcastle.ac.uk (J.R.); A.O.Odeniyi2@newcastle.ac.uk (A.O.); z.akhter@newcastle.ac.uk (Z.A.); 2 Department of Social Work, Education and Community Wellbeing, Northumbria University, Newcastle-Upon-Tyne, Tyne and Wear NE7 7XA, UK; tim.rapley@northumbria.ac.uk

**Keywords:** diet, physical activity, behaviours, determinants, pregnancy, child-bearing age, African, migrant, maternal overweight, maternal obesity

## Abstract

Dietary and physical activity behaviours during preconception and in pregnancy are important determinants of maternal and child health. This review synthesised the available evidence on dietary and physical activity behaviours in pregnant women and women of childbearing age women who have migrated from African countries to live in high income countries. Searches were conducted on Medline, Embase, PsycInfo, Pubmed, CINAHL, Scopus, Proquest, Web of Science, and the Cochrane library. Searches were restricted to studies conducted in high income countries and published in English. Data extraction and quality assessment were carried out in duplicate. Findings were synthesised using a framework approach, which included both a priori and emergent themes. Fourteen studies were identified; ten quantitative and four qualitative. Four studies included pregnant women. Data on nutrient intakes included macro- and micro-nutrients; and were suggestive of inadequacies in iron, folate, and calcium; and excessive sodium intakes. Dietary patterns were bicultural, including both Westernised and African dietary practices. Findings on physical activity behaviours were conflicting. Dietary and physical activity behaviours were influenced by post-migration environments, culture, religion, and food or physical activity-related beliefs and perceptions. Further studies are required to understand the influence of sociodemographic and other migration-related factors on behaviour changes after migration.

## 1. Introduction

Dietary and physical activity (PA) behaviours play a central role in the health and wellbeing of women and their children. This is especially important during preconception and in pregnancy. The preconception period represents a crucial time when dietary and PA behaviours could help prepare the body and ensure the accumulation of sufficient nutrient stores for healthy pregnancies in the future [1]. Maternal nutrition during pregnancy influences foetal growth and development and sets a foundation for long-term health for both mother and child [2]. Pregnant women are particularly vulnerable to inadequate nutrition due to the high nutrient and energy demands of pregnancy [3,4]. Proper foetal growth and development requires an appropriate nutritional intake at each stage of pregnancy, alongside adequate energy levels [5]. The World Health Organization (WHO) has established dietary guidelines and recommendations for intakes of various nutrients, to help improve health outcomes and prevent deaths in women and children worldwide [6]. The WHO also recommends that adults aged 18–64 years should do at least 150 min of moderate or 75 min of vigorous PA a week; or an equivalent combination of both [7]. Their definition of PA includes any leisure time activities (e.g., walking, dancing, gardening, hiking, and swimming), transportation (e.g., walking or cycling), occupational (i.e., work), household chores, playing, games, sports, or any planned exercise. These guidelines are applicable for all adults irrespective of gender, race, ethnicity, or income level [7]. Engaging regularly in moderate PA helps maintain a healthy weight, increases the chances of having a healthy pregnancy, and minimises the risk of poor pregnancy outcomes [8,9].

Behaviours can be shaped by a combination of intrinsic and extrinsic factors, such as personal preferences, cultural values, religion, and physical or social environments [10,11]. They can also be modified to variable extents by sociodemographic factors such as ethnicity, age, socioeconomic status, education, and income [12,13,14]. Although individuals respond differently to various factors, people in certain situations are seen to exhibit similar behaviours. Studies on migrant populations have shown that migration from one country to another is associated with changes in dietary and PA behaviours [15,16,17,18,19]. Most often, migrant populations tend to adopt the norms of the new host country, a phenomenon referred to as acculturation [20]. The impact of acculturation on dietary and PA behaviours may manifest as positive or negative changes. Findings from populations migrating from low-or-middle-income countries (LMICs) to high-income-countries (HICs) have shown that dietary behaviours in their home countries are usually healthier than those in HICs [20,21,22]. However, these tend to change overtime, converging towards (or becoming worse than) those of the host country population [20,22]. Changes include the adoption of less healthy dietary behaviours (e.g., overreliance on convenience foods and increased consumption of snacks and sweets) and sedentary lifestyles associated with minimal physical exertion [23,24,25]. Studies on PA report lower activity levels amongst migrant populations in HICs, especially in women [26,27,28,29].

Migration also influences health status and health outcomes. Disparities in health outcomes between migrant and majority populations in HICs are widely reported. A number of studies report that migrant women from African countries have greater risks of adverse pregnancy outcomes such as stillbirth, perinatal mortality, caesarean section, and preterm birth [30,31,32,33]. While the reasons for these differences are multifactorial, it is worth recognising that small changes in behaviours can have a substantial impact on population health [34]. Behavioural changes as a result of migration may offer explanatory theories and opportunities to improve maternal and child health outcomes. 

Robust synthesis of available evidence on dietary and PA behaviours amongst African pregnant women or women of childbearing age living in HICs is lacking. A better understanding of these behaviours could inform the development of sustainable interventions to improve health and pregnancy outcomes in this population. This systematic review aimed to synthesise the available evidence on dietary and PA behaviours, and determinants of these, amongst pregnant and childbearing age women who have migrated from African countries to live in HICs. 

## 2. Materials and Methods

A systematic review was conducted in accordance with the Preferred Reporting Items for Systematic Reviews and Meta-analysis (PRISMA) guidelines [35] and the protocol was registered on Prospero (CRD42017057562).

### 2.1. Study Identification

With the support of a librarian at Newcastle University, a comprehensive search strategy was developed for database searching (Appendix A). The search strategy was developed for a wider group of systematic reviews investigating weight status and weight-related behaviours in three populations: African migrant pregnant women, women of childbearing age, and children of African migrant women. This review focuses specifically on the studies on dietary and PA behaviours in pregnant women and women of childbearing age. Search terms and subject headings were developed for Medline and translated across eight other electronic databases: Embase, PsycInfo, Pubmed, CINAHL, Scopus, Proquest, Web of Science, and the Cochrane library. Searches were restricted to studies published between 1 January 1990 and 26 February 2018–1990 represents the start of a significant peak period of migration from African countries to HICs [36]. Database searches were completed in February 2018. Reference list and citation searches were completed in March 2018.

### 2.2. Inclusion and Exclusion Criteria

Inclusion criteria were: (1) primary research studies (qualitative and quantitative), (2) human participants, (3) pregnant women or women of childbearing age, (4) women who have migrated from an African country to live in a HIC, (5) studies conducted in HICs, (6) studies published in English language, and (7) studies reporting on at least one of the following: dietary behaviours, PA behaviours, or determinants of dietary and/or PA behaviours. Studies including “Black” women with no specification of country of origin were excluded, because these may have included women who have not migrated from African countries. Studies including African women living in HICs as refugees or asylum seekers were also excluded because they had been forcibly displaced and are therefore living in HICs under different circumstances from those of African women who migrated.

### 2.3. Study Selection and Screening

All studies resulting from the search were imported into EndNote Version X8. Titles and abstracts of a random 10% sample of all identified papers were independently screened by three authors (LN, JR, and NH), who reached a consensus on study eligibility and inclusion. Titles and abstracts for the remaining 90% of identified papers were then screened by one author (LN) and double screened by two other authors (AO and ZA). The full texts of all studies that met the inclusion criteria for this review were retrieved and screened by one author (LN); and then divided amongst the other five authors for double screening. Additional studies were identified by hand-searching the reference lists of all included studies and performing citation searches on Google Scholar. Any inconsistencies from the screening of full texts were resolved by discussion.

### 2.4. Data Extraction and Quality Assessment

A data extraction form was developed and piloted among three authors (LN, NH, and JR), for two studies. The final data extraction form included the title of the study, journal title, publication year, host country, country of origin of African women and outcomes reported. The quality of quantitative studies were assessed using the “Quality Assessment Tool for Observational Cohort and Cross-Sectional Studies” [37]; and the “Critical Appraisal Skills Programme (CASP) checklist” [38] was used for qualitative studies. Quality assessments were carried out in duplicate and any inconsistencies were resolved by discussion. Quality rating was judged as good, fair, or poor. A study was rated “good” if it was deemed to have a minimal risk of bias and its results were considered valid. A “fair” rating meant that the study was susceptible to some bias although not enough to invalidate its results. "Poor" quality referred to studies with major limitations and significant risk of bias. 

### 2.5. Data Synthesis

A framework synthesis approach was used, which offers a structured method to organise and analyse data [39], and is particularly useful to integrate qualitative and quantitative evidence in meta-synthesis. This method involves the utilisation of an a priori framework to code data, which can then be modified to reflect the evidence reported in the included studies [40]. The framework developed for this review was informed by background literature, and consisted of a list of a priori themes including dietary behaviours, PA behaviours and factors that influence dietary and PA behaviours (determinants). The determinants were sociodemographic factors (e.g., maternal age, parity, level of education, socioeconomic status (SES) income, and marital status); migration-related factors (e.g., duration of residence, age at arrival in HICs, and environmental factors); culture and religion; health status (e.g., stress); other health behaviours (e.g., smoking and alcohol consumption); pregnancy-related factors (e.g., pregnancy status and gestational age); and nutrition- or PA-related knowledge, beliefs, and perceptions. The framework was adapted throughout the process of data synthesis to incorporate and reflect themes that emerged from the data. This framework provided a matrix onto which relevant data from the included studies were coded. Relevant data included narratives from results sections, figures, data tables, and supplementary materials. 

The framework synthesis process involved two authors (LN and NH) and the following stages: familiarisation with the data, identification of a thematic framework, indexing, charting the data into the framework matrix, and mapping and interpretation. The familiarisation stage was an iterative process where all included studies were read several times, while marking portions of data with relevant information. Codes were then applied to fragments of text with information that was relevant to the review. Coding also involved making notes on questions to consider during the analysis process. The codes were then assessed for similarities and differences, and clustered together around similar concepts. Included studies were checked again to ensure that no new codes could be generated from the data. Any new themes that emerged were assessed to establish whether they were in fact new, or subgroups related to the existing a priori themes. In the final stage, the themes were used to explore patterns and relationships within the data. 

## 3. Results

**Figure 1:**
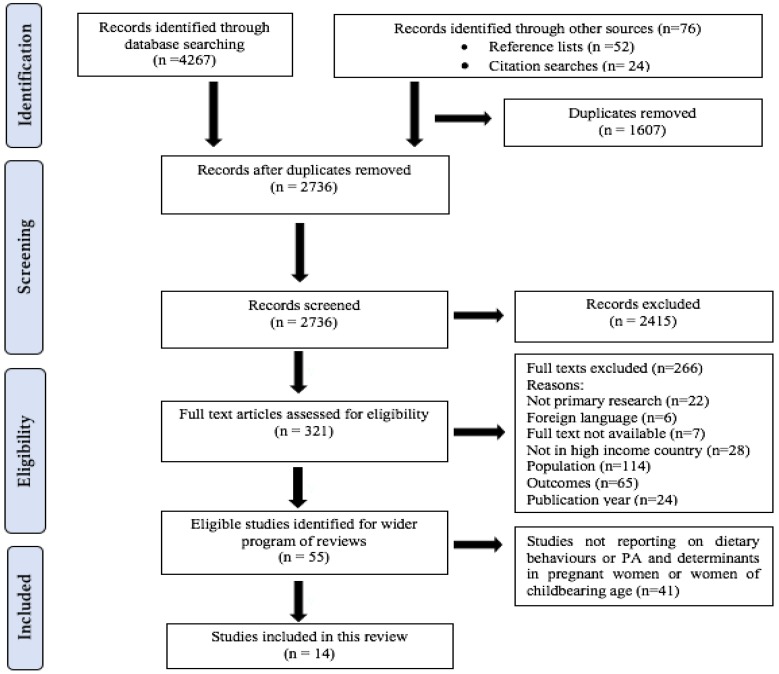
Systematic review PRISMA flow diagram.

The search strategy identified a total of 4343 citations (Figure 1). Exclusion of duplicates, initial screening of titles and abstracts and screening of full-texts against inclusion criteria left 55 potentially eligible studies. A further 41 studies were excluded from this review as they did not address dietary and PA behaviours in pregnant women or women of childbearing age and only reported data on weight status of women and children. These studies will inform additional systematic reviews relating to the wider research programme. Fourteen studies were included in this review.

### 3.1. Description of Included Studies

The characteristics and methodological quality of the 14 included studies are presented in Table 1. There were ten quantitative [41,42,43,44,45,46,47,48,49,50] (*n* = 9 cross-sectional and *n* = 1 longitudinal) and four qualitative [51,52,53,54] studies (*n* = 3 *focus* group discussions, *n* = 1 interview). Most of the studies were conducted in European countries (*n* = 2 each in Italy [42,43] and Spain [48,50]; *n* = 1 each in England [46], Ireland [44], the Netherlands [54], Norway [53], Sweden [51], and France [49]); two in Australia [41,47] and one each in Canada [52] and Israel [45]. Two main population groups were included in the studies: women from North and North-East African countries including Morocco, Somalia, Ethiopia, Egypt, Algeria, and Eritrea; and women from Sub-Saharan Africa (SSA) including Nigeria, Ghana, Ivory Coast, Benin, Niger, Mali, and Senegal. The number of participants ranged from 7–80 in the qualitative studies and 22–587 in the quantitative studies. A total of 1538 women were included, out of which 269 were pregnant. Pregnant women were included in four studies [42,44,49,52] and three of these reported their gestational ages. One study included women in their first trimester (<8 weeks) [42], one included women in their second and third trimesters (gestational age not reported) [44] and one included women in their third trimester (32–40 weeks) [49]. Eight studies reported how long the women had lived in HICs, which ranged between 2–35 years. Twelve studies included only African populations [41,43,44,45,47,48,49,50,51,52,53], while two [42,46] included women from other ethnic groups living in the same HIC for comparison. Quality assessment of the studies found eight “good” quality studies and six of “fair” quality; no included studies were deemed to be poor quality. 

### 3.2. Dietary Behaviours

Nine studies [42,44,45,46,47,48,49,50,53] reported dietary behaviours (Table 2). These included dietary intakes, dietary patterns, and food practices. Studies reporting on dietary intakes assessed energy, macronutrients and micronutrients using different methods of assessment including food-frequency questionnaires, 24 h recalls, face-to-face interviews, and researcher-developed questionnaires. Studies reporting dietary patterns assessed whether African women maintained their traditional dietary behaviours or adopted ‘Western-style’ dietary behaviours after migration. Studies reporting food practices described any factors relating to the production and consumption of food, such as cooking methods, meal planning, diet restrictions, and eating out of home.

#### 3.2.1. Dietary Intakes

Dietary intakes were assessed in four studies [44,45,48,50]. Results were presented as mean (SD) and percentage of total energy (%TE) derived from macronutrients. Two studies [44,45] also reported on nutrient supplement usage and three reported on nutrient inadequacies [44,45,50]. As there were limited comparison groups reported in the studies, this review compared the dietary results reported for African women with WHO recommendations [6,55].

##### Energy and Macronutrient Intakes

Table 3 shows the reported mean intakes of energy and macronutrients in pregnant women and women of childbearing age; and WHO recommended intakes. Five macronutrients were analysed, including carbohydrate, protein, total fat, dietary fibre, and cholesterol. Only one study [44] in Ireland reported intakes in pregnant women, which included total energy, dietary fibre, and %TE from carbohydrate, protein, and fat [44]. The study did not compare the women’s mean intakes with any recommendations but found that their %TE values were compliant with Irish national guidelines. When compared with WHO reference ranges, mean intakes of total energy and dietary fibre were low, while %TE were higher for protein and fat. 

Carbohydrate intakes in women of childbearing age were higher in two studies [45,48] compared to WHO recommendations, while intakes of dietary fibre were lower. Results for protein, fat and cholesterol were contradictory; while one study showed higher intakes than recommended by the WHO, the other study found lower intakes. Only one study reported %TE in women of childbearing age and all values were within the WHO reference ranges. No participants reported taking any nutrient supplements.

##### Micronutrient Intakes

A total of 15 micronutrients (Table 4) were reported in 3 studies [44,45,48], one of which included pregnant women [44]. Pregnant women’s intakes of vitamins A, B12, C, selenium, iron, and iodine were seen to be compliant with National Irish dietary guidelines, although iron intakes were less compliant than the other micronutrients (86.4% vs. 100%). Compared with WHO guidelines, intakes of vitamins A, B12, C, D, selenium, sodium, and iodine were higher than recommended. The study also reported inadequate intakes of calcium, folate, and vitamin D. Inadequate intakes of calcium, folate, and iron were seen following WHO recommendations, but not vitamin D. 

Two studies [45,48] found lower intakes of iron and higher intakes of vitamin C in women of childbearing age, compared to WHO reference ranges. Results for calcium and zinc were contradictory—intakes were higher in one study [48] and lower in the other [45]. Only one study [45] reported on vitamin B12, magnesium, folate, and phosphorus; while another [48] reported on vitamin E, sodium and folic acid in women of childbearing age. Intakes of vitamin E, sodium, magnesium, and phosphorus were higher than recommended, while those of folate and vitamin B12 were lower. folic acid intakes were within the WHO reference range.

#### 3.2.2. Dietary Patterns

Dietary patterns were explored in eight studies [42,44,45,46,48,49,50,53], one included pregnant women [44]. The studies described the extent to which the women’s usual dietary habits had changed after migration, the food groups they consumed and the kinds of food they had adopted from their host countries. No study reported that the women had completely adopted the dietary patterns of their host countries. African women were seen to preserve some of their traditional dietary behaviours after migration; the extents of which varied across studies. Dietary patterns differed according to how strictly the women adhered to their traditional foods. Three patterns of adherence were identified—strict, flexible, and limited. Adopting foods from the host country was reported for all three dietary patterns; and such foods were usually eaten at breakfast and lunch. Examples of these included processed meats, pizza, cereals, fish, chips, sandwiches, snacks, candies, and soft drinks. 

Two studies [44,49] (both in pregnant women) found that African migrant women strictly maintained their traditional dietary patterns. In these groups of women, snacking between meals was uncommon and the consumption of Western-style processed foods such as sausages, sugar-sweetened cereals, cheese, chilled desserts, cakes, biscuits, and pastries was very low [44]. One of the studies [44] described the composition of the women’s daily diets, which predominantly consisted of rice, other grains, tubers, fruits, vegetables, fish, meat, and grains. 

In contrast, two studies [45,53] observed that African migrant women (non-pregnant) adhered less strictly to their traditional African foods, although these were still consumed quite often. Other Western-style foods such as sandwiches, snacks, candies, and soft drinks were also consumed. One study presented the compositions of the women’s diets; they had higher intakes of grains and simple sugars; and lower intakes of vegetables, meat and fish. 

Dietary patterns characterized by a limited consumption of traditional foods were identified in two studies [48,50] (both non-pregnant women). Local African foods were rarely eaten by these groups of women and their dietary patterns were similar to those seen in most HICs. Both studies defined these as “Western” dietary patterns. The women in one of the studies consumed foods high in carbohydrates and fats and had high intakes of alcohol; as opposed to their traditional diets which contained more protein, less fats, and less alcohol [48].The most commonly consumed food groups were dairy products, meat, fish, raw vegetables, and fruits [48,50].

Two studies [42,46] compared food consumption patterns in African women with those of women from other ethnic groups. One of these included pregnant women [42]. Post-migration consumption of snacks was significantly higher in African women (North Africa—4.5% and SSA—51.5%) compared to Central and Eastern European women (8%) [42]. On the other hand, consumption of fruits and vegetables was significantly lower in African women (North Africa—45.5% and SSA—36.4%; Central and Eastern Europe (62.5%) [42]. In another study [48], African women were seen to have similar energy intakes with Spanish women, and higher intakes of carbohydrate, protein, cholesterol, dietary fibre, and alcohol. 

#### 3.2.3. Food Practices

The food practices reported were mostly reported as “coping mechanisms” used by African women, to facilitate their adaptation into their new food environments. These were behaviours related to shopping, cooking, and eating practices [45,47,49,54]. 

##### Shopping and Cooking Practices

African women reported searching for African foods from local shops and markets, to allow them to continue with their normal African diets [47]. These foods were usually not available or in short supply. Examples of local foods that were difficult to locate included African vegetables (e.g., sweet potato leaves, cassava leaves, amaranth, and pumpkin leaves), black-eye beans, maize flour, camel milk, and cocoyam products [47]. Women also reported substituting local foods with similar items found in HICs, in order to replicate their traditional dishes, such as replacing one type of meat for another [47]. Some unfamiliar host-country food items such as asparagus and figs were tried and rejected due to taste [47]. Although African women adopted some food items from their host countries, they showed preference for their traditional cooking and seasoning methods. African women pointed out unpleasant differences between host-country and traditional foods [53,54]. These differences related to taste, texture, and cooking methods. HIC foods were commonly described as “tasteless”, which usually meant the lack of salt or spices. Three studies [45,47,53] reported that women usually added salt or other local spices to HIC foods, to make them more palatable. One study in Norway [53] reported that African women seasoned fish differently and preferred frying rather than poaching, as was commonly done in Norway.

##### Eating Practices

Changes in African women’s meal plans were reported in two studies [45,47]. While some women reported taking breakfast more frequently after migration, some reported doing so less frequently, and others completely stopped [45,47]. A lack of structure in eating plans was mainly attributed to the nature of the women’s work lives. Pregnant women in one study reported restricting themselves from certain HIC foods during pregnancy (e.g., processed meats) and mostly consuming African vegetables [49]. Women reported a higher frequency of eating out of their houses since they migrated [47]. 

### 3.3. Determinants of Dietary Behaviours

Five studies provided data on the factors influencing dietary behaviours in African immigrant women [45,47,52,53,54], one included pregnant women [52]. The populations included in four studies were women from North and North East African countries including Somalia, Ethiopia, Morocco, Algeria, Egypt, and Eritrea. Only one study included women from SSA. The studies were conducted in Canada, Australia, Amsterdam, Israel, and Norway. 

The determining factors influencing the women’s dietary behaviours included five of the seven a priori themes (sociodemographic factors; migration-related factors; culture and religion; pregnancy-related factors, and nutrition-related knowledge, beliefs, and perceptions) and one data-driven theme (competing priorities). No data were reported for the a priori themes health status and other health behaviours. Some of the themes presented played a dual role as both barriers and facilitators to women’s behaviours.

#### 3.3.1. Sociodemographic Factors

Very limited data was available for sociodemographic factors. Only the influence of maternal age was reported in one paper [45] which reported a negative correlation with the consumption of dairy products, fats, simple sugars, and soft drinks (*p* < 0.001). No data were available for the influence of parity, maternal level of education, socioeconomic status (SES), income or marital status on dietary behaviours.

#### 3.3.2. Migration-related Factors

These determinants were all related to the new environments that African women lived in after migrating to HICs, specifically the new natural, food, living and work environments.

Natural Environment 

One study reported the influence of the weather in HICs on the women’s dietary behaviours [52]. Organic food and homegrown fruits, vegetables, and grains were more readily available throughout the year in their countries of origin compared with HICs.

Food Environment 

The food environment in HICs was reported both as a barrier and a facilitator to healthy dietary behaviours. The constant availability of food was a facilitator as this brought a level of food security in the women’s households compared with their countries of origin [47]. However, the availability of cheap and unhealthy convenience foods was a barrier to healthy dietary behaviours, which influenced women to eat out more, cook less, and consume more snacks [47,52,53,54]. 

Living Environment 

African women’s food choices were often influenced by the preferences of family members with whom they lived [53,54]. Most women reported that their husbands preferred traditional foods, which influenced the continuity of traditional dietary habits in their households. Other women reported that while their husbands preferred traditional foods, their children preferred foods adopted from the host country. This resulted in the women cooking separate meals for their husbands and children.

Work Environment

Living and working in HICs made the women’s schedules very busy and also increased the amount of time they spent away from home, which decreased their frequency of cooking at home and increased their frequency of eating snacks and take-away foods [45,52,53]. The women’s work schedules also influenced how often they were able to take breakfast or eat together with their families. 

No data were reported for the influence of the women’s duration of residence or age at arrival in HICs on their dietary behaviours.

#### 3.3.3. Culture and Religion

African women emphasised the importance of culture to their dietary behaviours and traditional food habits [45,47,53,54]. Food played a key role in showing hospitality and it was normal in their culture to serve food in large quantities. The women expressed pride in their African cuisine, highlighting the importance of using traditional spices to enhance taste. Cultural and religious festivals were commonly cited as reasons to cook traditional dishes. Religious rules also played a role on food choices and shopping patterns, especially for Muslim women who did not eat pork or had to determine whether food was “halal” (adheres to Islamic law) before consumption. Culture and religion were seen to reinforce the women’s efforts to maintain their traditional dietary behaviours.

#### 3.3.4. Pregnancy Status

Reasons for eating unhealthy foods during pregnancy included tiredness, long work hours, pregnancy stress, and a lack of support from family and friends in HICs [52]. Pregnant women also reported following their traditional behaviours and restraining from certain food items in order to avoid having a baby that was “too large”.

#### 3.3.5. Nutrition-Related Knowledge, Beliefs and Perceptions

In two studies, African women believed that HIC foods were healthier than their traditional foods because they were less dense and contained less oil and sugar [53], while traditional dishes were described as oily, spicy, high in calories, and fattening—all of which they attributed to poor nutrition [54]. Other women felt that some HIC foods were “lacking in nutrients” [53]. An example of this was vegetables—the women believed that boiling vegetables (as was commonly done in their host countries) made them watery and less nutritious; unlike frying, which helps preserve nutritional value and maintain crispiness. 

#### 3.3.6. Competing Priorities

Other priorities which typically prevented women from preparing home-made meals included going to work, attending school, looking after children, and managing day-to-day tasks at home [47,53,54]. Foods adopted from HICs were frequently cooked because they were considered less time-consuming to prepare and enabled women to serve warm meals on busy days. These factors also influenced eating outside the home or ordering food online.

### 3.4. PA Behaviours

Five studies [41,43,45,46,50] reported PA behaviours in women of childbearing age (Table 5); there were no studies in pregnant women. Two studies included North and North East African women living in Australia and Israel; two included women from West and SSA living in Spain and Italy; and one did not specify the region of origin of the African women living in England. PA behaviours were assessed and defined differently, as shown in Table 5. All methods of PA assessment were self-reported. Three studies included all-African populations [43,45,50], while two included women from other ethnic groups for comparison [41,46]. 

Findings from the all-African population studies reported conflicting results. Two studies of North East African women in Spain [50] and West African women in Israel [45] showed that the majority (65.9% and 72%, respectively) did not exercise regularly, and 49% of the women in Israel walked for less than 30 min a day. However, another study reported that 87% of women from SSA living in Italy exercised more than 3 times a week [43]. 

Studies including comparison groups of non-African women also reported conflicting data. One study [41] reported no significant different in physical inactivity between North African women and Australian women (OR 1.07, 95% CI 0.60–1.88), while another group of African women (countries of origin not specified) were significantly less inactive (OR 0.69, 95% CI 0.51–0.94). A second study [46] showed that Black African women in England were less likely to exercise more than 3 times in 4 weeks than White women (OR physical inactivity 2.16, 95% CI 1.71–2.75). 

### 3.5. Determinants of PA Behaviours

Four studies explored the determinants of PA in African migrant women [45,51,52,54]; one included pregnant women [52]. The populations in all four studies were women from North and North East African countries, including Somalia, Morocco, Ethiopia, and Eritrea. No studies included women from West Africa or SSA. The studies were conducted in Canada, Sweden, Amsterdam, and Israel. 

The same framework was used as described for determinants of dietary behaviours (Section 3.2). Four out of seven a priori themes (sociodemographic factors, migration, culture/religion, and PA-related knowledge, beliefs, and perceptions) were reported in the included studies. No data was reported for health status, other health behaviours, and pregnancy-related factors. Participants’ responses were based on their interpretations of PA, which mostly referred to organised PA (e.g., going to the gym, cycling, or attending fitness classes) as opposed to habitual PA like walking and household chores. Only one theme (migration-related factors) included factors related to habitual PA. 

#### 3.5.1. Sociodemographic Factors

Findings on sociodemographic factors were limited to income and marital status. No data were available for maternal age, parity, level of education, or measures of SES additionally to income.

Income 

Income was reported as a barrier to PA in one study [52]. The women reported that they lacked financial resources to enrol in gyms or fitness classes that suited their needs. 

Marital Status 

One study described that married and cohabiting women reported their daily schedules to be very busy, leaving them with no time for extra activities other than everyday chores. 

#### 3.5.2. Migration-Related Factors

All four studies reported on migration-related factors and these were centred around the same environmental factors as dietary behaviours.

Natural environment 

The cold weather in HICs was reported as a barrier to PA in three studies [51,52,54]. The warm climate in their home countries enabled women to engage in outdoor activities like walking to the market. Meanwhile, outdoor activities in HICs were avoided due to the cold climate, except in the warmer seasons. The weather also influenced the means of transportation the women used during the winter as they preferred motorised rather than active transportation such as walking. North African women described how they were accustomed to staying indoors and sleeping in on rainy days, and only left the house if they had reason to (e.g., work or taking children to school) [51].

Built environment

Built environments in HICs presented a barrier to PA in that the streets around the women’s neighbourhoods usually either lacked sidewalks or had high volumes of traffic [51,54]. The wide availability of transport facilities (e.g., buses and trains) also significantly reduced the amount of time that the women spent walking [51,54]. The women felt that their environments “back home” were more conducive to being physically active [52] where they usually walked long distances to buy groceries and household supplies [51].

Living environment

African women described the houses where they lived in HICs as relatively small [54], and they had more household appliances which reduced their daily PA related to household chores [51,54]. However, women from North and North East Africa also reported that they were used to living with extended family members who all participated in household chores, whereas this was not the case in HICs which increased the amount of time required to complete chores and limited the spare time they had for exercise [51]. 

Work environment

Only one study included findings on African women’s work environments in HICs which tended to promote sedentary activity [45].

No study reported on the influence of the women’s duration of stay in HICs or their age at arrival.

#### 3.5.3. Culture and Religion

Cultural and religious factors played a role on women’s ability to exercise, especially women from predominantly Muslim African countries whose culture did not encourage women to mix with men in public spaces and there was a lack of female only centres [51,54]. Women described that their traditional outfits were not suitable for PA and the possibility of people watching while they exercised was an additional barrier to PA [51]. Participation in PA relied on the activities meeting their cultural needs, such as having an informal leader from a similar cultural background, being accompanied by other women, or being able to dress in their traditional attires [51].

#### 3.5.4. PA-Related Knowledge, Beliefs and Perceptions

African women showed an understanding for the need of PA and acknowledged that it is important to health and well-being. Participation in leisure-time or health-related PA was a concept women only became familiar with after migration. For example, women described how PA was normally incorporated into their daily lives in their countries of origin, so they were not familiar with the concept of walking for health or just walking for the sake of it [51]. This was also viewed as a facilitator to PA by women who had experienced positive health outcomes from such activities. Some women believed that leisure-time activities were meant for children [51]. A lack of familiarity was also expressed with some organised physical activities such as cycling or swimming and many had never learned to cycle or swim [54]. 

#### 3.5.5. Competing Priorities

Other responsibilities competing for African women’s time such as family commitments, work, school, and household chores were common barriers to PA [45,51,54]. Living and working in HICs was described as “very busy” and “stressful”; unlike in their countries of origin where most women didn’t have to work. 

## 4. Discussion

This review aimed to synthesise the evidence on dietary and PA behaviours, and determinants of behaviours, among pregnant women and women of childbearing age who have migrated from African countries to live in HICs. Data available for macronutrient intakes were conflicting. Micronutrient analyses suggest low levels of folate, calcium, and iron in pregnant women; while sodium intake was more than twice as high as recommended levels. Deficiencies in multiple micronutrients are a reflection of poor diets and are associated with pregnancy complications and haematological consequences, which may increase the risk of haemorrhage and death during pregnancy [56,57]. A high sodium intake increases the risk of metabolic disorders and pregnancy complications such as preeclampsia [58]. Future intervention research could prioritise improving the diet quality, especially micronutrients, for this population of women. 

Findings on dietary patterns showed that no participants completely changed their traditional dietary practices to those in HICs. Rather, dietary patterns were bicultural, with an overlap between HIC and traditional dietary practices. Three overarching patterns were defined according to how much the women adhered to their traditional dietary practices. These were either strict, flexible, or limited. Evidence of adopting host-country behaviours was seen across all three dietary patterns. Examples of adopted “unhealthy” dietary behaviours included increased frequency of snacking, high consumption of processed foods, high intakes of sweets and sweet drinks, cooking less, and eating out more often. Similar behaviours have been observed amongst migrant populations in other reviews [23,59]. A few healthy behaviours were also adopted, such as an increased consumption of fish, fruits, and vegetables; and taking breakfast more regularly [45,53].

Dietary behaviours were shaped by interrelating factors that fell into six main themes: sociodemographic characteristics, migration-related factors, culture and religion, pregnancy, nutrition-related knowledge, beliefs and perceptions, and competing priorities. Post-migration environments had the highest number of factors that shaped the women’s dietary behaviours. Religious and cultural beliefs predominantly influenced women from Muslim countries. Major facilitators to maintaining traditional dietary practices included cultural and religious beliefs, having African community and social networks, the availability of ethnic shops in HICs and participants’ perceptions of HIC foods. African women highlighted the importance of spiciness and taste in food; and showed a preference for traditional cooking methods. On the other hand, the likelihood of adopting host-country dietary practices was increased by factors such as the abundance of cheap, unhealthy convenience foods in HICs, busy lifestyles and not having enough time to cook African foods. The findings of this review fit in the model of dietary acculturation proposed by Satia-Abouta et al. [60], which shows that dietary changes are governed by sociodemographic, cultural, and environmental factors. Similar influences on dietary behaviours have also been reported in other reviews [23,61]. It is also possible that the process of change to Westernised behaviours could have started before migration, due to the nutrition transition occurring in LMICs. The nutrition transition is characterised by urbanisation and a shift from less energy dense diets and more active lifestyles to a higher consumption of fatty foods and sedentary lifestyles [62]. While the majority of evidence in this review shows that HIC environments contributed unhealthy dietary practices, other studies have found the contrary. For example, a study conducted in Belgium [63] reported increased consumption of healthy foods following migration. This study suggested that exposure to the host-country culture increased nutrition knowledge through media, friendships, and work environments; which led to the adoption of healthier eating habits.

Findings on PA behaviours were inconsistent, although there was a general sense of low levels of physical inactivity amongst women who had migrated from African countries. Inconsistency in results was mainly due to the difference in methods used to assess PA as well as different outcomes reported. The extent of behaviour change after migration may also vary according to country of origin and time of migration, so even individuals belonging to the same ethnic group may be at different levels of the acculturation process. This may explain some of the conflicting results presented in the studies. Determinants of PA behaviours were clustered around five main themes: sociodemographic characteristics, migration-related factors, culture, and religion, PA-related knowledge, beliefs and perceptions, and competing priorities. Culture, religion, and post-migration environments played the biggest role on women’s PA behaviours in the evidence reported. Cultural and religious beliefs were seen to prohibit participation in public activities, especially amongst women from Muslim countries. Migration to HICs was also shown to influence a more sedentary lifestyle, unlike the environmental and living conditions in the women’s countries of origin which were more favourable to PA. Although women were not familiar with several concepts of PA, participating in leisure-time activities was regarded as a positive adopted behaviour, which women enjoyed, provided the activities were organised to meet the women’s cultural customs.

This systematic review is the first to report robust evidence synthesis on African migrant women’s dietary and activity behaviours. Methodological strengths include the thoroughness of the search, which involved multiple databases plus supplementary searches, and duplication of all screening, data extraction and quality assessments. An in-depth framework synthesis was carried out to ensure that available evidence, as well as gaps in available evidence, were explicitly captured. Many studies excluded from this review either assessed dietary and PA behaviours in both women and men, but did not provide separate analyses for women, or included “Black” women but did not specify their countries of origin. These partly account for the limited number of relevant studies included in the review. The inclusion of mainly cross-sectional quantitative studies in this review also reflects the types of studies available. Studies predominantly included women from North African countries, with fewer women from West and Sub-Saharan Africa; and there may be different determining factors across African countries which could not be explored in this review. A major limitation across studies was the reliance on self-reported responses which may introduce recall bias, associated with inaccuracies in recollecting past events. Dietary intakes or PA levels may have been over- or under-reported, thereby not giving a true representation the women’s behaviours.

There was a paucity of data on dietary and PA behaviours in pregnant women. In addition, data were not available for several a priori factors which were drawn from evidence base of determinants of dietary and PA behaviours in other populations. These include sociodemographic factors (maternal age, parity, maternal education, and socioeconomic status), migration-related factors (duration of residence, age at time of migration, and immigrant status), health status, other health behaviours (smoking or alcohol consumption) and pregnancy-related factors (gestational age or pregnancy specific health conditions such as gestational diabetes). Further studies are required to explore the influence of these factors on women’s dietary and PA behaviours. Other factors such as feelings of inclusion or exclusion; or a sense of identity or belonging with either the host country or the participants’ countries of origin were also not explored in the included studies. Further studies are needed to understand the influence of these factors, as they could potentially modify the women’s behaviours.

There was also a paucity of data which directly compared migrant and host-country populations. Further research is needed to understand differences and commonalities in factors underlying their dietary and PA behaviours. 

## 5. Conclusions

The findings of this review highlight the need to understand the role of acculturation on dietary and PA behaviours amongst women who have migrated from African countries to live in HICs. The contradictory evidence of migration-related factors highlights that acculturation is a complex and multifactorial process. As both barriers and facilitators were reported in this data, the process of acculturation could either play a protective or detrimental effect on health by influencing positive or negative health behaviours. The continuation of some traditional practices is an indication of the value placed on cultural habits and the need for culturally sensitive approaches in understanding post-migration behavioural changes. Future research is needed to explore the evidence gaps identified in this review to gain a deeper understanding of the factors that bring changes in behaviours after migration. 

## Figures and Tables

**Table 1:** Characteristics of included studies.

Author	Host Country	Study Type	Participants’ Regions (Countries) of Origin and Sample Size	Participant Ages (Years)	Duration of Residence in HIC (Years)	Outcome(s) Measured	Study Quality
Persson et al., 2014 [51]	Sweden	Qualitative	East Africa (Somalia), *n* = 26	17–67	Mean 11.56 (Range 1–23)	Determinants of PA behaviours	Good
Quintanilha et al., 2016 [52] ^1^	Canada	Qualitative	North East Africa (Eritrea, Ethiopia, Somalia), *n* = 80	NR	NR	Determinants of dietary behaviours Determinants of PA behaviours	Fair
Dassanayake et al., 2011 [41]	Australia	Quantitative	North Africa, Other Africa (countries not specified), *n* = 122 Comparison group—Australia (*n* = 6460)	NR	NR	PA behaviours	Good
Delisle et al., 2009 [50]	Spain	Quantitative	West Africa (countries not specified), *n* = 130	<30–≥45	<6–>11	Dietary behaviours PA behaviours	Good
Garnweidner et al., 2012 [53]	Norway	Qualitative	North Africa (Algeria, Egypt, Morocco); North East Africa (Somalia), *n* = 7	25–60	2–35 (50% population <10 years)	Dietary behaviours Determinants of dietary behaviours	Good
Casali et al., 2015 [42] ^1^	Italy	Quantitative	North Africa and Sub-Saharan Africa (countries not specified), *n* = 66	Mean (SD) 31.8 (7.3)	NR	Dietary behaviours	Fair
Dominguez et al., 2008 [43]	Italy	Quantitative	Sub-Saharan Africa (Ivory Coast, Ghana, Nigeria, Mali, Benin), *n* = 23	Mean (SD) 29.9 (8.3)	NR	PA behaviours	Good
Lindsay et al., 2014 [44] ^1^	Ireland	Quantitative	West Africa (Nigeria), *n* = 52	Mean (SD) 32 (6.3)	Mean (SD) 7.5 (3.2)	Dietary behaviours	Fair
Regev et al., 2012 [45]	Israel	Quantitative	North East Africa (Ethiopia), *n* = 53	Mean (SD) 32.0 (6.0)	Mean (SD) 13.8 (5.8) 13.8 ± 5.8	Dietary behaviours Determinants of dietary behaviours PA behaviours Determinants of PA behaviours	Good
Smith et al., 2011 [46]	England	Quantitative	Black African (region or countries not specified), *n* = 587	16–34	NR	Dietary behaviours PA behaviours	Fair
Nicolaou et al., 2012 [54]	Netherlands	Qualitative	North Africa (Morocco), *n* = 22	27–59	<10–≥21	Determinants of dietary behaviours Determinants of PA behaviours	Good
Renzaho 2006 [47]	Australia	Quantitative	Sub-Saharan Africa (countries not specified), *n* = 111		NR	Dietary behaviours Determinants of dietary behaviours	Good
Gil et al., 2005 [48]	Spain	Quantitative	West Africa (countries not specified), *n* = 130	<30–≥45	Mean (SD) 8.2 (6.9)	Dietary behaviours PA behaviours	Fair
Roville-Sausse 1998 [49] ^1^	France	Quantitative	Sub-Saharan Africa (Senegal, Mali, Niger), *n* = 129	18–41	<10	Dietary behaviours Determinants of dietary behaviours	Fair

Footnote: PA—Physical activity NR—Not reported, ^1^ Study includes pregnant women.

**Table 2:** Studies porting on dietary behaviours and determinants of dietary behaviours.

Author	Host Country	Population Countries of Origin	Dietary Outcomes Measured	Data Collection Methods
Delisle et al., 2009	Spain	West Africa (countries not specified), *n* = 130 No comparison group	Dietary patterns after migration	Interviews, FFQ
Garnweidner et al., 2012	Norway	North Africa (Algeria, Egypt, Morocco); North East Africa (Somalia), *n* = 7 No comparison group	Dietary patterns after migration, determinants of dietary behaviours	Interviews
.Casali et al., 2015 ^1^	Italy	North Africa and Sub-Saharan Africa (countries not specified), *n* = 66 Comparison groups (Non-African): Central and Eastern Europe (*n* = 24)	Dietary patterns after migration, comparison of food group consumption patterns with non-African ethnic groups	Interviews, Questionnaires
Quintanilha et al., 2016 [52] ^1^	Canada	North East Africa (Eritrea, Ethiopia, Oromo, Somalia), *n* = 80 No comparison group	Determinants of dietary behaviours	FGD
Lindsay et al., 2014 [44] ^1^	Ireland	West Africa (Nigeria), *n* = 52 No comparison group	Dietary intakes	Interviews, 24 h recall
Regev et al., 2012 [45]	Israel	North East Africa (Ethiopia), *n* = 53 No comparison group	Dietary patterns after migration, determinants of dietary behaviours	Interviews, 24 h recall
Smith et al., 2011 [46]	England	Black African (region or countries not specified), *n* = 587 Comparison group White Irish, *n* = 466	Comparison of food group consumption patterns with non-African ethnic groups	Survey, Questionnaires
Nicolaou et al., 2012 [54]	Netherlands	North Africa (Morocco), *n* = 22 No comparison group	Determinants of dietary behaviours	FGD
Renzaho 2006 [47]	Australia	Sub-Saharan Africa (countries not specified), *n* = 111 No comparison group	Dietary patterns and food practices after migration, determinants of dietary behaviours	Interviews
Gil et al., 2005 [48]	Spain	West Africa (countries not specified), *n* = 130 No comparison group	Dietary intakes	Interviews, FFQ
Roville-Sausse 1998 [49] ^1^	France	Sub-Saharan Africa (Senegal, Mali, Niger), *n* = 129 No comparison group	Dietary patterns and food practices after migration, determinants of dietary behaviours	Not reported

Footnote: PA—PA, FFQ—Food-frequency questionnaires, FGD—Focus Group Discussions, ^1^ Study includes pregnant women.

**Table 3:** Daily energy and macronutrient intake and %TE in pregnant women and women of childbearing age.

Energy and Macronutrient	Reported Intakes				WHO Recommended Intakes		
	Mean (SD)—Pregnant Women [44]	Mean (SD)—Women of Childbearing Age [45,48]	%TE Pregnant Women [44]	%TE Women of Childbearing Age [45]	Mean Intake in Pregnancy	Mean Intake in Women of Childbearing Age	%TE ^2^ (both Pregnant Women and Women of Childbearing Age)
Energy (Kcal)	2393 (784)	NR	51.9	N/A	2750 ^1^	N/A	N/A
Carbohydrate (g)	NR	163.2 (63.6) 273.6 (67.0)	19.4	65	N/A	130	55–75
Protein (g)	NR	33.8 (16.1) 107.2 (24.6)	33.3	13.5	N/A	46	10–15
Total fat (g)	NR	25.3 (10.2) 85.7 (22.7)	447.4	22.8	N/A	<70	15–30
Cholesterol (mg)	NR	66 447.4	NR	66	N/A	<300 mg/day	<300 mg/day
Dietary fibre (g)	20.0 (9.8)	8.6 (4.4) 21.3 (6.0)	NR	NR	28	25	N/A

Foot note: ^1^ Reference for second and third trimesters used to correspond with gestational age of included women; ^2^ Same recommendations used for both groups of women as no increased requirements are reported in pregnancy; NR—Not reported, N/A—not applicable.

**Table 4:** Daily micronutrient intakes in pregnant women and women of childbearing age.

Micronutrient	Mean Intakes		WHO Recommended Intakes	
	Mean (SD) Pregnant Women	Mean (SD) Women of Childbearing Age	Mean Intakes in Pregnancy [6]	Mean Intakes in Women of Childbearing Age
Vitamin A (μg) [44,48]	1448 (1886) [44]	878.2 (944) [48]	800	500
Vitamin B12 (μg) [44,45]	6.8 (5.2) [44]	0.75 (0.66) [45]	2.6	2.4
Vitamin C (mg) [44,45,48]	224.6 (130.4) [44]	175.3 (92.3) [48] 70 (51) [45]	55	45
Vitamin D (μg) [44]	5.1 (6.5) [44]	NR	5	N/A
Vitamin E (mg) [48]	NR	14.0 (4.1) [48]	N/A	7.5
Calcium (mg) [44,45,48]	726.6 (425.8) [44]	1074 (344) [48] 277 (152) [45]	1500–2000	1000
Selenium (μg) [44]	77.9 (37.0) [44]	NR	30	N/A
Sodium (mg) [44,48]	4646 (2130) [44]	4266 (1250) [48]	<2000	<2000
Folate (μg) [44,45]	308.4 (141.9) [44]	129 (58) [45]	600	400
Iron (mg) [44,45,48]	14.0 (5.8) [44]	18.2 (3.9) [48] 12.8 (6.6) [45]	27	24
Iodine (μg) [44]	247.6 (163.0) [44]	NR	200	N/A
Magnesium (mg) [45]	NR	228 (51) [45]	NR	220
Folic acid (g) [48]	NR	399.5 (109) [48]	NR	400
Zinc (mg) [45,48]	NR	13.0 (3.1) [48] 4.1 (0.7) [45]	NR	4.9–9.8
Phosphorus (mg) [45]	NR	635 (15) [45]	NR	580

Footnote: NR—Not reported; N/A—not applicable.

**Table 5:** Studies reporting on PA and determinants of PA.

Author	Host Country	Population Countries of Origin	PA ^1^ Outcome Measured (Including Definitions of PA)	Method of PA Assessment
Dassanayake et al., 2011 [41]	Australia	North Africa, Other Africa (countries not specified), *n* = 122 Comparison group–Australia (*n* = 6460)	frequency and amount of time spent on PA relating to sport and fitness in the past 2 weeks	Self-report
Persson et al., 2014 [51]	Sweden	East Africa (Somalia), *n* = 26	Determinants of PA behaviours	Self-report
Quintanilha et al., 2016 [52] ^1^	Canada	North East Africa (Eritrea, Ethiopia, Oromo, Somalia), *n* = 80	Determinants of PA behaviours	Self-report
Delisle et al., 2009 [50]	Spain	West Africa (countries not specified), *n* = 130	Frequency of exercise (activities not defined)	Self-report
Dominguez et al., 2008 [43]	Italy	Sub-Saharan Africa (Ivory Coast, Ghana, Nigeria, Mali, Benin), *n* = 23	Frequency of PA per week. PA defined as moderate or strenuous exercise sufficient to induce sweating including any sport, brisk walking, or doing housework at least once a week for >30 min	Self-report
Regev et al., 2012 [45]	Israel	North East Africa (Ethiopia), *n* = 53	Daily exercise, PA at work, time spent walking per day	Self-report
Smith et al., 2011 [46]	England	Black African (region or countries not specified), *n* = 587	Moderate or vigorous PA of at least 30 min in the last 4 weeks	Self-report
Nicolaou et al., 2012 [54]	Netherlands	North Africa (Morocco), *n* = 22	Determinants of PA behaviours	Self-report
Gil et al., 2005 [48]	Spain	West Africa (countries not specified), *n* = 130	Regular practice of exercise (activities not defined)	Self-report

Footnote: PA—PA, ^1^ Study includes pregnant women.

## Appendix A

### Medline Search Strategy

1. africa*.mp. [mp = title, abstract, original title, name of substance word, subject heading word, keyword heading word, protocol supplementary concept word, rare disease supplementary concept word, unique identifier, synonyms]
2. exp Africa/
3. African Continental Ancestry Group/
4. (foreign born adj6 black*).mp.
5. ethnic minorit*.mp.
6. 1 or 2 or 3 or 4 or 5
7. “Emigrants and Immigrants”/
8. migrant*.mp.
9. immigrant*.mp.
10. emigrant*.mp.
11. 7 or 8 or 9 or 10
12. exp Women/
13. Mothers/
14. exp Child/
15. infant/ or infant, newborn/
16. (baby or babies).mp. [mp = title, abstract, original title, name of substance word, subject heading word, keyword heading word, protocol supplementary concept word, rare disease supplementary concept word, unique identifier, synonyms]
17. toddler*.mp.
18. offspring.mp.
19. young child*.mp.
20. kindergarten.mp.
21. preschool*.mp.
22. pre-school*.mp.
23. 12 or 13 or 14 or 15 or 16 or 17 or 18 or 19 or 20 or 21 or 22
24. exp Obesity/
25. exp Overweight/
26. bodyweight.mp.
27. body weight changes/ or weight gain/
28. maternal obesity.mp.
29. obes*.mp.
30. adiposity.mp.
31. body fat*.mp.
32. fat mass.mp.
33. exp Body Composition/
34. body mass index/ or body size/
35. weight.mp.
36. (obes* adj6 pregnan*).mp.
37. (weight adj6 pregnan*).mp.
38. (bmi adj6 pregnan*).mp.
39. (obes* adj6 prepregnan*).mp.
40. (obes* adj6 pre-pregnan*).mp.
41. (weight adj6 prepregnan*).mp.
42. (weight adj6 pre-pregnan*).mp.
43. (bmi adj6 prepregnan*).mp.
44. (bmi adj6 pre-pregnan*).mp.
45. Eating or diet or food or intake
46. Exercise or activity or PA
47. Pediatric Obesity/
48. Fetal Macrosomia/
49. large for gestational age.mp.
50. weight for height.mp.
51. infant nutrition.mp.
52. (child* adj2 nutrition).mp.
53. infant feeding.mp.
54. (child* adj2 feeding).mp.
55. exp Breast Feeding/
56. complimentary feeding.mp.
57. exclusive breastfeeding.mp.
58. 24 or 25 or 26 or 27 or 28 or 29 or 30 or 31 or 32 or 33 or 34 or 35 or 36 or 37 or 38 or 39 or 40 or 41 or 42 or 43 or 44 or 45 or 46 or 47 or 48 or 49 or 50 or 51 or 52 or 53 or 54 or 55 or 56 or 57
59. Knowledge or beliefs or perceptions
60. Behaviour* or behavior* or attitude* or habit*
61. Determinant* or factor* or influence*
62. 59 or 60 or 61
63. 6 and 11 and 23 and 56 and 62
